# Prolonged electrocardiography registration does not lead to increased diagnosis of atrial fibrillation in pulmonary embolism patients, but sex affects generic health-related quality of life: Findings from a randomized clinical trial

**DOI:** 10.1097/MD.0000000000032197

**Published:** 2022-12-02

**Authors:** Eli Westerlund, Awat Fili, Emma Svennberg

**Affiliations:** a Karolinska Institutet, Department of Clinical Sciences, Danderyd University Hospital, Stockholm, Sweden; b Karolinska Institutet, Department of Medicine, Karolinska University Hospital Huddinge, Stockholm, Sweden.

**Keywords:** atrial fibrillation, pulmonary embolism, quality of life, sex

## Abstract

Pulmonary embolism (PE) is the third most common cause of cardiovascular death. Atrial fibrillation (AF) is the most common arrhythmia and is commonly detected in patients after embolic stroke. In this study, we set out to study if undiagnosed AF could be detected in patients with PE. In survivors of PE, persisting symptoms and exercise limitations are common. The disease burden of PE on quality of life (QoL) has received scarce attention. The primary aim was to study the prevalence of AF in patients with newly diagnosed PE. The secondary aim was to study QoL in PE patients.

**Methods:**

Patients with newly diagnosed acute PE were randomized 1:1 to long-term electrocardiogram (ECG) screening for AF (handheld ECG or ECG patch) or standard-of-care. The study participants were asked to complete RAND-36 questionnaires upon inclusion.

**Results:**

In total 89 PE patients (mean age 74.6 years) were included, and 40 out of these patients were randomized to AF screening. The study was terminated early due to futility when analysis 1 year after inclusion did not find any patients with newly detected AF.

RAND-36 showed that QoL was affected in PE patients. Interestingly, sex differences were found; women had a significantly lower QoL in the dimensions of vitality (*P* = .006), general health (*P* = .039), and mental health (*P* = .041).

**Conclusion:**

Screening for AF in PE patients did not yield a significant proportion of new cases. QoL is more affected in female patients with PE, and increased awareness of this is suggested.

## 1. Introduction

Pulmonary embolism (PE) is the third most common cause of cardiovascular death after myocardial infarction and stroke.^[1]^ Globally, the yearly incidence of PE ranges from 39 to 115/100 000 of the population.^[2]^ The reported yearly incidence of PE in Sweden is 60/100 000.^[3]^ PE counts for approximately 25% to 30% of cases within the venous thromboembolic disease spectrum.^[4,5]^

Silent or overt deep vein thrombosis (DVT) is considered to be the cause of PE in most cases.^[1,5]^ However, recent studies have shown variable results of DVT presence ranging from 36% to 93% in patients with PE.^[6]^ Such data suggest that the cause might be more multifaceted than the established belief of DVT causing PE.

Atrial fibrillation (AF) is the most common sustained arrhythmia with a prevalence of approximately 3%.^[7]^ Untreated AF is associated with a 5-fold increased risk for developing embolic ischemic stroke.^[3,8]^ In one older Swedish autopsy report examining 693 patients with AF, 5% had right atrial thrombosis and 3.7% had thrombi in both atria.^[9]^ Hence, there is a possibility that thrombi formed in the right atrium during AF could dislodge into the pulmonary circulation, causing PE. A previous retrospective cohort study showed a 12 times increased risk of PE during the first 6 months after AF diagnosis,^[10]^ whereas a recent registry study demonstrated no significant association between AF and the development of PE after adjustment for oral anticoagulant therapy.^[11]^ To study if patients with PE have silent AF, we initiated the SAFE-PE study to screen for AF. The gain for screening PE patients for AF would be continuous anticoagulation treatment instead of a shorter course of anticoagulants of 3 to 6 months, which is common routine for many patients with uncomplicated PE. Indeed, patients with continuous anticoagulant therapy have been shown to have a significantly lower risk of both venous and arterial thromboembolism.^[3,12,13]^

Patient related outcome measures aim to evaluate disease states from the patient perspective. The disease burden of PE on Quality of Life (QoL) has received scarce attention, even though every second PE patient has perfusion defects in the lungs 6 months after their PE diagnosis.^[14]^ One year after diagnosis every second PE patient suffer from persisting symptoms and physical exercise limitations.^[15]^

This study was conducted to screen for AF in PE patients and to study the quality of life in PE patients with and without AF.

## 2. Methods

### 2.1. Patients

Consecutive patients with an acute PE were identified at the Department of Radiology or at the outpatient follow-up at Danderyd Hospital, Stockholm, Sweden.

Inclusion criteria: Acute PE diagnosis within 3 months, without a prior diagnosis of AF; fulfilling requirements for life-long oral anticoagulant (OAC) therapy in case of AF diagnosis (CHA_2_DS_2_-VASc 2 points for men and 3 points for women).

Exclusion criteria: Known diagnosis of AF; not eligible for OAC therapy in case of AF diagnosis; and known cancer with recent surgery, undergoing chemotherapy, or with life expectancy <1 year.

Participants were randomized 1:1 to be screened for AF or receive standard-of-care as a control group. The randomization was performed after the patients were assigned as study participants by using sealed opaque envelopes. Participants who got randomized into the screening arm were screened for AF for 4 weeks using a validated, handheld electrocardiogram (ECG) device (Zenicor EKG-2, Sweden) twice daily for 30 seconds or in case of palpitations or with a continuous ECG patch (Philips Biotel) for 5 days. The participant registrations were analyzed for AF weekly and when disagreements or difficulties in interpreting the recording occurred, an independent senior cardiologist was consulted. Only patients with heart palpitations or irregular heart rate upon follow-up visit received additional 12-lead ECGs.

Patient characteristics and survival status was assessed by reviewing the electronic medical records of all patients at inclusion and 1 year after PE diagnosis.

The primary aim was to study the prevalence of screening-detected AF in patients with newly diagnosed PE treated at Danderyd Hospital, Stockholm, Sweden. The secondary aim was to study the quality of life in PE patients.

Ethical permit for the study was approved by the regional ethics board in Stockholm 2017/829-31/1 and oral and written consents were given by all participants.

### 2.2. Objective examinations

PE was confirmed by computer tomography or perfusion scintigraphy.

All pulmonary and lobar artery emboli were categorized as central PE. Segmental and subsegmental embolisms were categorized as peripheral PE. In case of several embolisms on different levels the most centrally located embolism was counted. All participants had a standardized transthoracic echocardiogram.

### 2.3. Quality of life questionnaire

The study participants were asked to complete a QoL questionnaire during the time of inclusion:

RAND-36,^[16]^ a QoL measure with a recall period of 4 weeks and with a scale ranging from 1 to 100, where 100 means that the patient is asymptomatic. The RAND-36 quality of life questionnaire has been used previously in the Swedish general population.^[17]^ RAND-36 is similar to Short Form 36 Health Survey Questionnaire (SF-36)^[18,19]^ but does not require a license. It consists of 36 questions covering 8 different concepts of well-being: general health perceptions, role limitation due to physical health, role limitation due to emotional problems, bodily pain, vitality, mental health, social functioning, and emotional well-being. We calculated the unweighted physical health composite score by adding the sub scores of general health perceptions, role limitation due to physical health, bodily pain and vitality and divided the sum by four.

For the mental health composite score we added the sub scores of role limitation due to emotional problems, mental health, social functioning, and emotional well-being and divided the sum by four.^[20]^

### 2.4. Statistical analyses

Using a power analysis with two-sided equality based on the study by Ng et al^[21]^ we assumed that AF would be detected in 30% of the screened population. We assumed that our primary outcome, all-cause mortality, would be 46% after 5 years in participants with screening-detected AF, and 30% in those without screening. If oral anticoagulant therapy protects against 70% of the events and using a power of 80% and a Type 1 error rate of 5%, 364 participants would need to be included in each arm. To account for loss of follow-up we planned to include a total of 800 patients.

The inclusion of patients was halted due to the COVID-19 pandemic during March 2020. After the inclusion was halted an analysis of data gathered was performed. This analysis was not planned, and the study sample may be underpowered.

Nominal and dichotomous variables are presented as absolute numbers and proportions.

Continuous variables were assessed for normality using histograms and, Shapiro-Wilks’ test; presented as means and 95% confidence interval for normally distributed data and median and interquartile range for data with a skewed distribution. To compare the QoL between males and females and even between patients with central and peripheral PE, Mann–Whitney test was used for RAND-36 calculations. *P* values below .05 were considered statistically significant. We used SPSS (version 27.0) for all statistical analyses.

## 3. Results

In total, 89 patients (mean age 74.6, 38.2% women) with a confirmed acute PE were included between 2017 and 2020. Inclusion, RAND-36 assessments, and randomization were performed on average 17 days after the PE diagnosis. Forty patients were randomized to AF screening and 49 patients to standard of care (controls).

Patient characteristics at baseline (time of acute PE) are shown in Table 1. Hypertension was the most common comorbidity with 55 (61.8%) of the patients using antihypertensive treatment at the time of inclusion. Eighteen (20%) had a history of previous venous thromboembolism and 41 (46%) had an unprovoked PE event.

**Table 1:** Main baseline characteristics of the study patients.

All patients (n = 89)	
Male sex (n, %)	55 (61.8)
Age (mean, CI)	74.6 (73.2–76.0)
BMI (mean, CI)	27.4 (26.5–28.3)
Localization of PE	
- Pulmonary artery (n, %)	28 (31.5)
- Lobar artery (n, %)	32 (36)
- Segmental (n, %)	25 (28.1)
- Subsegmental (n, %)	4 (4.5)
Previous VTE (n, %)	18 (20)
Unprovoked PE (n, %)	41 (46)
Heart failure (n, %)	4 (4.5)
Hypertension (n, %)	55 (61.8)
Diabetes (n, %)	10 (11.2)
Previous stroke/TIA (n, %)	5 (5.6)
CHA_2_DS_2_VASc (median, ICR)	3 (2)
Active cancer (n, %)	3 (3.4)
Active/previous smoking (n, %)	39 (43.8)
Death (n, %)	4 (4.5)
Cause of death	
Pneumonia (n, %)	1 (1.1)
Lung fibrosis (n, %)	1 (1.1)
Malignancy (n, %)	1 (1.1)
Unknown (n, %)	1 (1.1)

CI = confidence interval, ICR = interquartile range, PE = pulmonary embolism, TIA = transient ischemic attack, VTE = venous thromboembolism.

After inclusion of 89 patients, the SAFE-PE study was paused due to the COVID-19 pandemic during which an early, unplanned, interim analysis was performed. As there were no patients with newly detected AF in the screening group it was deemed futile to continue inclusion in the trial.

Thirteen of the patients reported dyspnea and 3 had chest pain at the follow-up visit 4 to 6 weeks after the PE diagnosis. During the ECG screening no AF nor any other clinically actionable arrhythmias were detected. The cardiac ultrasound performed upon inclusion showed a mean pulmonary artery pressure of 42 mm Hg and 6 patients had a pulmonary artery pressure of >60 mm Hg.

All participants were followed 1 year after PE diagnosis. Four patients died during the first year after diagnosis. The patients that died all had peripheral PE, and PE was not determined as cause of death in any of the cases. Cause of death is listed in Table 1.

As shown in Table 2, the QoL was affected in PE patients. The roles of physical function and vitality were most affected among patients with PE. There was no significant difference in the RAND-36 QoL dimensions between central and peripheral PE patients. However, bodily pain was more common in patients with peripheral PE.

**Table 2:** RAND-36 levels in patients with central versus peripheral pulmonary embolisms. The levels are ranging from 0 to 100 with higher levels meaning better quality of life.

RAND-36			
	Central pulmonary embolism	Peripheral pulmonary embolism	
	Median (IQR)	Median (IQR)	p-level
Physical functioning	70.0 (45.0–85.0)	70.0 (35.0–87.5)	ns
Role physical	25.0 (0.0–75.0)	25.0 (0.0–75.0)	ns
Bodily pain	67.5 (45.0–90.0)	62.5 (32.5–88.8)	ns
General health	60.0 (40.0–75.0)	65.0 (42.5–72.5)	ns
Vitality	55.0 (40.0–70.0)	65.0 (45.0–77.5)	ns
Social function	62.5 (50.0–87.5)	62.5 (37.5–81.3)	ns
Role emotional	66.7 (33.3–100.0)	66.7 (0.0–100.0)	ns
Mental health	80.0 (60.0–92.0)	78.0 (60.0–92.0)	ns
Summary scores:			
Physical health	55.6 (32.5–81.3)	55.6 (27.5–80.9)	ns
Mental health	66.1 (45.8–87.3)	68.1 (35.6–87.7)	ns

ns = not significant IQR = interquartile range.

**Figure 1.**
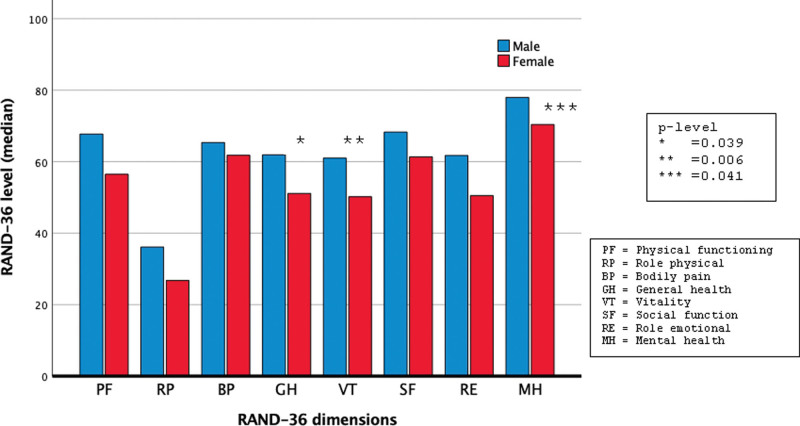
RAND-36 levels in female (red bars) and male (blue bars) patients with acute pulmonary embolism. Low RAND-36 level means low quality of life.

Interestingly, when comparing males and females with PE, women had a significantly lower QoL in the dimension vitality (*P* = .006), general health (*P* = .039) and mental health (*P* = .041), as shown in Figure 1. The summary scores for physical health showed that women had a 10% lower quality of life (women 49.4, men 60.3). A 10% lower summary score was also noted in women with regards to mental health compared to males (women: 61.8, men 75.8).

## 4. Discussion

The results of the present study concluded that reduced QoL is a frequent finding in patients with acute PE, regardless of a central or peripheral located PE. Women had significantly lower QoL regarding vitality, mental and general health compared to the men with PE. Screening for AF did not detect any new cases of the arrhythmia in the group randomized to prolonged heart rhythm monitoring.

In the present study, we excluded individuals with known AF and individuals with AF diagnosed at admission or during hospital stay. The aim of the initial, randomized study was to determine if screening for AF after PE would change standard of care, but no AF was detected amongst those randomized to screening for arrhythmias. A high background detection could be the reason for our low rates of detection.

Our results are in line with the result from the previous register study from our research group following 1.5 million randomly selected Swedish residents during 7 years where the study endpoint was a first-time event of PE. Of the residents 48.000 had AF, but there was no association between AF and PE if OAC treatment was considered.^[11]^ Another retrospective analysis of 2449 patients admitted for a clinically suspected PE concluded that the presence of AF did not increase the probability of PE in the setting where PE was suspected.^[22]^ In a subgroup analysis from the same study, patients with both chest pain and AF had a slightly increased probability of PE.

Patients with a reduced QoL and PE had an increased risk of long-term mortality, shown by Keller et al, following 101 German patients for 3.6 years.^[23]^ They used the Pulmonary Embolism Quality of Life, a disease specific and validated QoL questionnaire used for PE^[24,25]^ both upon presentation and 6 months after PE diagnosis. In their cohort 25% experienced post-PE impairment 6 months after their initial hospital visit, compared to 18% of the patients in our study only 1 month after PE diagnosis.

The larger FOCUS study published in CHEST in 2021 included 620 patients using the Pulmonary Embolism Quality of Life questionnaire at 3- and 12-months following PE. Interestingly, and in line with our findings, a sex difference was discovered showing a reduced QoL in women.^[26]^

Patients with peripheral PE experience more pain due to pleural effusion.^[27]^ Interestingly, in our study, patients with central and peripheral PE both experienced pain and had reduced QoL because of physical discomfort. There could be several explanations. First, PE patients often have predisposing factors, like pneumonia, recent surgery, etc which could cause pain. In our study, 54% of the patients had a provoking factor. Second, several patients with central PE also had peripheral PE that could potentially cause pleural effusion or lung infarction causing chest pain.

The women in our study showed a significantly larger reduction of vitality, mental and general health compared to the males. In the Tehran Lipid and Glucose Study >7000 individuals were examined for their QoL.^[28]^ The mean mental health related QoL summary scores were significantly lower in those with cardiovascular diseases (CVD) compared to those without CVDs, and the women with CVDs experienced an impaired mental health compared to the men with CVDs.

Limitations: the small sample size is a potential limitation of our study as we terminated the inclusion due to the COVID-19 pandemic during March 2020. As patients with COVID-19 infection is more prone to PE, screening for AF was considered less important. This early analysis was therefore not planned, and the study sample may be underpowered for futility.

As we only included patients with PE, assumptions about associations between AF and PE cannot be made. The fact that we excluded patients with AF upon presentation of the PE diagnosis may have influenced the result since none of the included patients had AF during the ECG screening. Also, the fact that the twice daily ECG registration only lasted 30 seconds may have influenced the results.

As we included the patients up to 90 days after PE diagnosis, they also had a variable time until the QoL questionnaire, which may have affected the results. Overall, the mean time from PE diagnosis was 17 days and most of the patients were included during the first month after PE diagnosis. Our findings apply to PE patients only and may not be valid in other populations. The decision to prolong oral anticoagulant therapy in patients with a first PE is complex and inter-and intra-variations between practices might occur. These factors might affect the generalizability of this study.

To conclude, female patients with PE have more affected QoL. There is scarce evidence regarding sex differences and life quality after PE. The sex difference in QoL from our study could be hypothesis generating for further clinical QoL trials among patients with PE.

Our results suggest that attention towards the awareness of functional limitations after PE, both in the acute setting and during follow-up, should be given.

The study was not powered to support AF screening in patients with PE, but the very low AF detection suggests against implementation of AF screening of all PE patients.

## Acknowledgments

The authors would like to thank research nurses Maja Månsson and Lena Gabrielsson from the Department of Clinical Sciences, Karolinska Institutet Danderyd Hospital, for support during the inclusion period and professor Håkan Wallén for scientific advice during the study period.

## Author contributions

**Conceptualization:** Eli Westerlund.

**Data curation:** Eli Westerlund.

**Formal analysis:** Eli Westerlund.

**Funding acquisition:** Emma Svennberg.

**Investigation:** Awat Fili.

**Methodology:** Emma Svennberg.

**Project administration:** Awat Fili.

**Resources:** Emma Svennberg.

**Software:** Eli Westerlund.

**Supervision:** Emma Svennberg.

**Validation:** Emma Svennberg.

**Writing – original draft:** Eli Westerlund.

**Writing – review & editing:** Eli Westerlund, Awat Fili, Emma Svennberg.
